# Sequential Anti-Cytomegalovirus Response Monitoring May Allow Prediction of Cytomegalovirus Reactivation after Allogeneic Stem Cell Transplantation

**DOI:** 10.1371/journal.pone.0050248

**Published:** 2012-12-13

**Authors:** Sylvia Borchers, Melanie Bremm, Thomas Lehrnbecher, Elke Dammann, Brigitte Pabst, Benno Wölk, Ruth Esser, Meral Yildiz, Matthias Eder, Michael Stadler, Peter Bader, Hans Martin, Andrea Jarisch, Gisbert Schneider, Thomas Klingebiel, Arnold Ganser, Eva M. Weissinger, Ulrike Koehl

**Affiliations:** 1 Department of Hematology, Hemostasis, Oncology and Stem Cell Transplantation, Hannover Medical School, Hannover, Germany; 2 Pediatric Hematology and Oncology, Johann Wolfgang Goethe-University, Frankfurt, Germany; 3 Institute of Human Genetics, Hannover Medical School, Hannover, Germany; 4 Institute of Virology, Hannover Medical School, Hannover, Germany; 5 Internal Medicine II, Johann Wolfgang Goethe-University, Frankfurt, Germany; 6 Institute of Pharmaceutical Science and Biostatistics, ETH Zürich, Switzerland; 7 Institute of Cellular Therapeutics, IFB-Tx, Hannover Medical School, Hannover, Germany; University of Palermo, Italy

## Abstract

**Background:**

Reconstitution of cytomegalovirus-specific CD3^+^CD8^+^ T cells (CMV-CTLs) after allogeneic hematopoietic stem cell transplantation (HSCT) is necessary to bring cytomegalovirus (CMV) reactivation under control. However, the parameters determining protective CMV-CTL reconstitution remain unclear to date.

**Design and Methods:**

In a prospective tri-center study, CMV-CTL reconstitution was analyzed in the peripheral blood from 278 patients during the year following HSCT using 7 commercially available tetrameric HLA-CMV epitope complexes. All patients included could be monitored with at least CMV-specific tetramer.

**Results:**

CMV-CTL reconstitution was detected in 198 patients (71%) after allogeneic HSCT. Most importantly, reconstitution with 1 CMV-CTL per µl blood between day +50 and day +75 post-HSCT discriminated between patients with and without CMV reactivation in the R+/D+ patient group, independent of the CMV-epitope recognized. In addition, CMV-CTLs expanded more daramtaically in patients experiencing only one CMV-reactivation than those without or those with multiple CMV reactivations. Monitoring using at least 2 tetramers was possible in 63% (n = 176) of the patients. The combinations of particular HLA molecules influenced the numbers of CMV-CTLs detected. The highest CMV-CTL count obtained for an individual tetramer also changed over time in 11% of these patients (n = 19) resulting in higher levels of HLA-B*0801 (IE-1) recognizing CMV-CTLs in 14 patients.

**Conclusions:**

Our results indicate that 1 CMV-CTL per µl blood between day +50 to +75 marks the beginning of an immune response against CMV in the R+/D+ group. Detection of CMV-CTL expansion thereafter indicates successful resolution of the CMV reactivation. Thus, sequential monitoring of CMV-CTL reconstitution can be used to predict patients at risk for recurrent CMV reactivation.

## Introduction

Reactivation of cytomegalovirus (CMV) remains one of the major complications after allogeneic hematopoietic stem cell transplantation (HSCT) [1], [2], [3]. The latent virus is controlled mainly by CMV-specific T cells (CMV-CTLs) in healthy persons, but in immune-compromised patients CMV reactivation occurs frequently due to impaired T cell reconstitution and post-HSCT immunosuppressive therapy. CMV reactivation, if not controlled, can lead to severe and multiple manifestations of CMV disease, such as CMV retinitis, gastroenteritis or pneumonia [4]. CMV disease is also associated with a high risk for bacterial or fungal infections [5] and development of graft-versus-host disease (GvHD) [6], [7]. Major risk factors for CMV reactivation include recipient CMV-seropositivity (R+), T cell-depletion (TCD) of the graft [2], [8], [9] and pre-established acute GvHD (aGvHD) [7], [10]. Monitoring CMV viral load by real-time polymerase chain reaction (PCR) or by pp65 expression in leukocytes is used to direct pre-emptive antiviral therapy to reduce the risk for CMV disease. The high sensitivity of PCR-based methods may invoke treatment of patients who do not need medication at that time, thus immunohistochemical detection of pp65 is still used in addition to PCR [11]. Pre-emptive therapy with ganciclovir (GCV) has significantly reduced incidence and severity of CMV disease, but has been associated with severe side effects, the primary ones being myelo- and/or nephrotoxicity [12].

Multimers, such as the tetrameric HLA epitope complexes (tetramer), are commonly used to monitor CMV-specific CTL reconstitution [13], [14], [15], [16], [17], [18], and can be used as a tool to analyze the reconstitution process. Although several studies have investigated CMV-CTL levels as a possible predictor for CMV reactivation, no clear protective threshold has yet been defined. While differences between patient cohorts and transplantation protocols may make the definition of a reliable threshold value for therapy initiation or withdrawal difficult, the HLA molecules and tetramer combinations used to detect CMV-CTLs may contribute to the variation observed to date in meaningful CMV-CTL levels. Monitoring time-points or time-frames of patients also differed in these studies, further complicating the interpretation of the results [14], [15], [16], [17]. To address these questions, we prospectively monitored the reconstitution of CMV-specific immune responses in 278 patients using 7 commercially available tetramers [15], [19] representing various CMV epitopes.

We hypothesized that monitoring using tetramers could be a valuable tool to individualize antiviral therapy, especially for patients at increased risk for developing multiple CMV reactivations. We analyzed factors that influence the CMV-CTL level, to assess the possibility of identifying the minimal number of CMV-CTLs required to provide protection against reactivation and with the intent of defining optimal monitoring time-point(s) for CMV-CTL reconstitution in HSCT patients.

## Design and Methods

### Ethics statement

Sample collection and analyses were part of an extended monitoring program conducted in the course of routine sampling for clinical follow-up. The study was approved by the University Hospital Ethics Committees (Hannover and Frankfurt, Germany), and is registered as #2906 with the Ethics Committee at the Hannover Medical School and as #50/07 with the Ethics Committee at the University Hospital Frankfurt. Written informed consent was obtained from all patients or legal guardians.

### Patient characteristics

Patients were transplanted between January 2006 and December 2010 in the Departments of Pediatric Hematology and Oncology and Internal Medicine in Frankfurt and the Department for Hematology, Hemostasis, Oncology and Stem cell transplantation at Hannover Medical School. This prospective study included all patients (n = 278) who had at least one HLA molecule corresponding to a set of 7 commercially available HLA-CMV tetramers (Coulter; USA). Patients were transplanted for leukemia, lymphoma, myelodysplastic or benign hematopoietic dysfunction syndromes or solid tumors in pediatric patients after the first relapse of the underlying disease. HLA-matched donors were available for 78% (n = 218/278) of patients, while 22% (n = 60/278) received grafts from mismatched donors. Bone marrow (BM; n = 35), cord blood (CB; n = 2), peripheral blood stem cells (PBSC; n = 220) or T cell-depleted (TCD) PBSC (n = 21) were used as grafts. T cell depletion was done *ex vivo* as described previously [20], [21] using good manufacturing practice (GMP), and reduced T cells in the graft by 13,000-fold compared to unselected PBSC and 800-fold compared to BM. Sixty-eight percent (n = 190/278) of the patients were CMV-seropositive (R+), and 73% (n = 139/190) of this group were transplanted from CMV-seropositive donors (D+), with the remaining 27% (n = 51/190) receiving grafts from CMV-seronegative donors (D−). Prior to HSCT, 88 recipients (32%) were CMV-seronegative (R−), of whom 56% (n = 49/88) received grafts from CMV-seronegative donors, while 44% (n = 39/88) were transplanted from CMV-seropositive donors. Patient and graft characteristics are summarized in Table 1. GvHD prophylaxis included OKT3 (Janssen-Cilag, Netherlands), thymoglobulin (Genzyme; USA) or antithymocyte globulin (ATG, Fresenius, Germany) in combination with cyclosporine A (CsA), mycophenolate mofetil (MMF) or methotrexate (MTX), respectively.

**Table 1:** Patient characteristics.

Patient cohort			n = 278
Age mean (range)			43.5 (2–72)
**Gender**			n
Female			119
Male			159
**Diagnosis**			n
ALL			41
AML			104
sAML			29
CLL			5
CML			9
MDS			35
solid tumors/lymphoma			31
other diseases with indication for HSCT			24
**CMV reactivations/CMV DeNovo Infection in the various**			
**CMV-serostatus recipient (R)/donor (D) group**			n/n
All patients			117/278
R+/D+			77/139
R+/D−			30/51
R−/D+			7/39
R−/D−			3/49
**Donor**			n
MRD			87
MUD			131
MMUD			40
MMRD			20
**Transplant**			n
BM			35
	CD34 *10^6^/kgBW (mean):	3.1	
	CD3 *10^6^/kgBW (mean):	25.8	
PBSC without TCD			220
	CD34 *10^6^/kgBW (mean):	13.4	
	CD3 *10^6^/kgBW (mean):	391.6	
PBSC with TCD			21
	CD34 *10^6^/kgBW (mean):	10.4	
	CD3 *10^6^/kgBW (mean):	0.03	
other*			2
**Matched tetramers per patient**			**n**
1 tetramer			102
2 tetramers			126
3 tetramers			49
4 tetramers			1
**CMV-epitopes**			n
HLA-A*0101 (pp50)			95
HLA-A*0201 (pp65)			156
HLA-A*1101 (pp65)			1
HLA-A*2402 (pp65)			53
HLA-B*0702 (pp65)			73
HLA-B*0801 (IE-1)			77
HLA-B*3501 (pp65)			41

ALL: acute lymphatic leukemia; AML: acute myeloid leukemia; sAML: secondary AML; CLL; chronic lymphoid leukemia; CML: chronic myeloid leukemia; MDS: myelodysplastic syndrome;

MRD: matched related donor; MUD: matched; MMUD: mismatched unrelated donor; MMRD: mismatched related donor; TCD: T cell depletion.

BM: bone marrow; PBSC: peripheral blood stem cell;

* cord blood.

### Detection of CMV infection/reactivation after allogeneic HSCT

Blood/serum samples from all patients were routinely monitored for CMV DNA load using PCR [18] or for pp65 expressing cells per 400,000 leukocytes in peripheral blood mononuclear cells (PBMNCs) using immunohistochemistry [13]. Pre-emptive antiviral therapy was initiated when a) the CMV DNA load increased by more than 0.5 log levels above the baseline, b) more than 2 pp65-expressing cells were present per 400,000 leukocytes in 2 consecutive tests or c) more than 5 pp65-expressing cells were present per 400,000 leukocytes in a single test. The initial therapy was ganciclovir (GCV; 5 mg/kg twice a day), which was adjusted according to the presence of pp65-expressing PBMNC or CMV DNA load [13].

### Flow cytometric quantification of CMV-CTLs

We monitored patients on days +30, +60, +90, +120 and +200 (all time-points +/−15 days) after HSCT. Patients who a) experienced CMV reactivation or b) at increased risk for reactivation due to increased immunosuppression were monitored weekly. Weekly monitoring was stopped when a) CMV reactivation was resolved or b) immunosuppression reduced. CMV-CTLs were quantified using HLA-CMV epitope tetramers as previously described [13], [18]. HLA-typing of patients and donors was conducted during preparation for HSCT via high-resolution multiplexed PCR [22].

**Figure 1:**
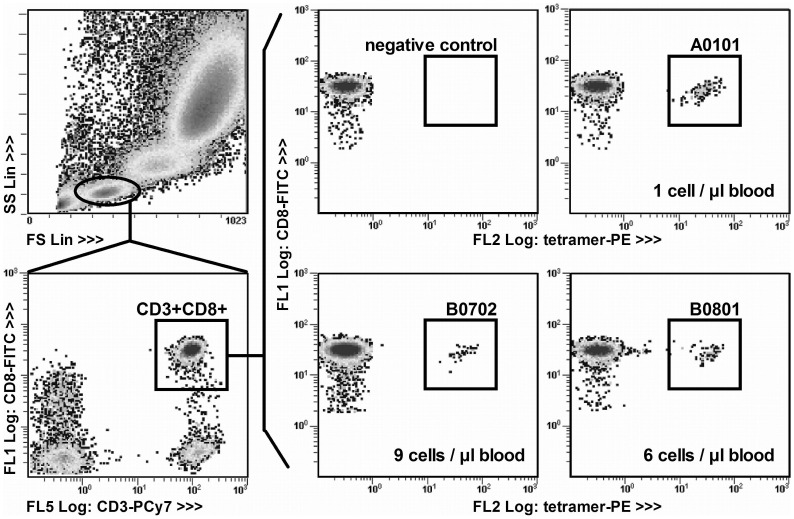
Gating strategy for detection of tetramer-stained CMV-CTLs. The gating hierarchy and tetramer positive cells in a patient expressing 3 of the HLA molecules represented in our tetramer set are shown. The lymphocytes were gated in the FS/SS quadrant (upper left) followed by the selection of CD3^+^CD8^+^-positive cells (lower left). Within the CD3^+^CD8^+^-positive population, the percentage of cells that bind each tetramer is determined in individual samples (200 µl whole blood each). Staining results using the negative control tetramer, HLA-A*0101 CMV tetramer, HLA-B*0702 CMV tetramer and HLA-B*0801 CMV tetramer are shown. The number of CMV-CTLs per µl blood is indicated in each tetramer plot.

CD3^+^CD8^+^ T cells were quantified using 100 µl of EDTA-anticoagulated blood stained with 10 µl CD3-PCy5/7-labelled anti-CD3 antibody (Beckman Coulter, Germany), 10 µl FITC-labelled anti-CD8 clone T8 antibody (Beckman Coulter, Germany), 20 µl PE-labelled anti-CD4 antibody (Beckman Coulter, Germany) and fluorescent beads (FlowCount™ beads, Beckman Coulter, Germany) added according to the manufacturer's instructions. CMV-CTLs were quantified using the following commercially available set of PE-labeled tetramers: HLA-A*0101-VTEHDTLLY, pp50 amino acids (aa) 243–255; HLA-A*0201-NLVPMVATV, pp65 aa 495–503; HLA-A*1101-ATVQGQNLK, pp65 aa 501–509; HLA-A*2402-QYDPVAALF, pp65 aa 341–349; HLA-B*0702-TPRVTGGGAM, pp65 aa 417–426; HLA-B*0801-ELRRKMMYM, IE-1 aa 199–207; HLA-B*3501-IPSINVHHY, pp65 aa 123–131 (all: Beckmann-Coulter, Germany). Each tetramer corresponding to a HLA molecule expressed in the patient was measured separately and required 200 µl EDTA-anticoagulated blood. Aliquots were stained with 10 µl αCD3, 10 µl αCD8 and 5 µl tetramer. One negative control was performed per patient using 200 µl of EDTA-anticoagulated blood and 5 µl negative control tetramer, to which none of the cells should specifically bind to, provided by the manufacturer (Beckman Coulter, Germany). All samples were labeled for 30 min at room temperature (RT), followed by erythrocyte lysis (15 min, RT) and fixation. The gating strategy and staining results are shown in Figure 1. At least 10,000 lymphocytes, 1000 CD3^+^CD8^+^ cells and 10 tetramer-positive cells were counted for each tetramer-stained sample. At least 10,000 lymphocytes and 1000 CD3^+^CD8^+^ cells were counted for each negative control tetramer-stained sample and in addition 1100 fluorospheres were counted for each CD3^+^CD8^+^ T cell quantification.

Absolute numbers of CMV-CTLs were calculated using fluorescent beads (FlowCount™ beads, Beckman Coulter, Germany) in a single-platform, no-wash analysis according to the manufacturer's directions. Briefly, samples were washed and analyzed, after erythrocyte lysis (VersaLyse, Lysing Solution; IO 3, Fixative Solution 10×; Beckman Coulter, Germany), on a FC500 flow cytometer (Beckman Coulter, Germany) [13] [18]. The absolute number of CMV-CTLs in a sample was calculated by subtracting negative-control-tetramer-binding cells from CMV-CTLs binding only to CMV-tetramers. Table 1 summarizes the tetramers corresponding to HLA-molecules present in patients. Quality constraints were determined in our previeous studies [13] [18] and we determined that only whole blood samples containing at least 50 CD3+CD8+ T-cells per µl blood gave reliable and between centers reproducible results to cytometrically detect multimer-positive cells at a reliable event rate as detailed above. The detection limit is 0.05 multimer-positive cells per µl blood using these quality constraints. Application of these quality constraints allowed to include 92% (n = 1712) of 1861 samples in this tri-center trial (Table S1). Low CD3^+^CD8^+^ counts below 50/µl blood in 149 samples (8%) led to the exclusion of generated data from this analysis.

### Data management and statistical analysis

CMV-CTL data were collected and stored in a mySQL database. The general purpose PHP5 (Personal Home Page tools 5; open source license) scripting language was used for queries in the database. The mean number of CMV-CTLs from all tests using one tetramer was calculated for each patient to evaluate whether monitoring using single tetramers resulted in similar CMV-CTL counts in patients with detectable immune responses. For patients monitored with more than 1 tetramer, the CMV-CTL counts obtained for each tetramer were calculated individually after HSCT. Clinical data were correlated with CMV-CTL reconstitution. The influence of the presence or absence of CMV-CTL on the occurrence of CMV reactivation and *vice versa* was evaluated. CMV-CTL reconstitution was analyzed at different thresholds: namely >0, ≥1, 3, 5, 7 or 10 CMV-CTL per µl blood). CMV-CTL levels before and after 1^st^ CMV reactivation were counted, and the resulting slope between this 2 time-points was calculated. For the determination of CMV-CTL expansion after CMV reactivation, the following patient/donor pairs were excluded: a) CMV-seronegative patients who were transplanted from seronegative donors and did not develop *de novo* CMV infections during follow-up; b) patients who died before day +100, c) patients with early relapse of the underlying disease by day +100 and d) patients for whom sampling could not be achieved prior to and after CMV reactivation. Statistical and Kaplan-Meier analyses were performed with GraphPad Prism 4 and 5 (GraphPad Software, San Diego, USA). Graphs were plotted using GraphPad Prism 4 and 5). P-values≤0.05 were considered significant, and the significance test applied is indicated for all p-values in the figure legends.

## Results

### CMV-CTL levels vary depending on the tetramer used for detection, on HLA-molecules expressed and on occurrence of CMV reactivation

**Figure 2:**
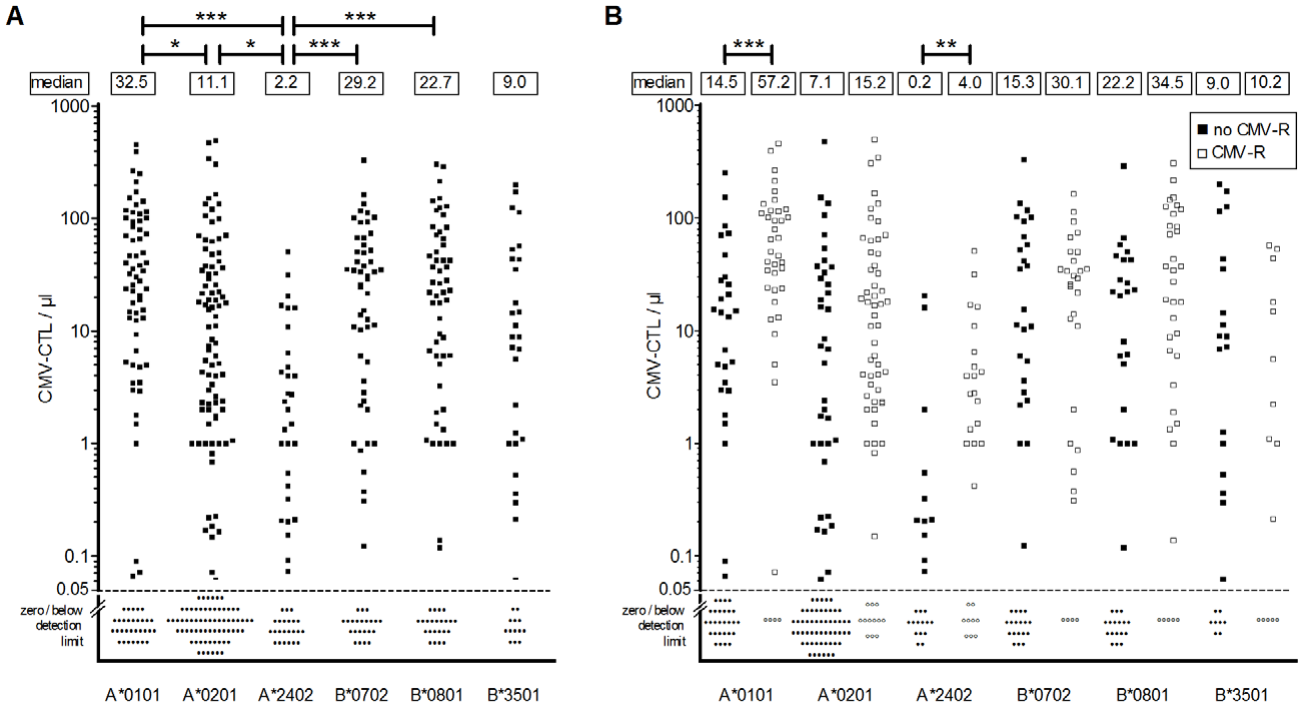
CMV-CTL levels vary depending on the HLA-epitope tetramer used for detection and the occurrence of CMV-reactivation. (**A**) Median circulating CMV-CTL levels in our patient cohort are shown for six different HLA-restricted CMV epitopes monitored by commercially available HLA-epitope tetramers. Data are shown for all patients with a detectable CD3+/CD8+ T cell response (CMV-CTLs >0 per µl blood) during the first year following HSCT, and summarized as mean values of CMV-CTLs calculated for each patient (filled squares). P-values were calculated using the Kruskal-Wallis test followed by Dunn's multiple comparison. Patients not mounting a response (0 CMV-CTL per µl blood) or with cell counts below the detection limit are denoted below the dashed line. (**B**) Median CMV-CTL levels for each HLA-restricted CMV epitope are shown for each patient who experienced (empty squares, CMV-R) or did not experience (filled squares, no CMV-R) CMV reactivations. The median CMV-CTL levels from the entire cohort are shown in the box above each group. No results are shown for the HLA-A*1101 tetramer, since only one patient was monitored. * *p*≤0.05, ** *p*≤0.01, *** *p*≤0.001.

CMV-CTL reconstitution was observed in 71% (n = 198/278) of the patients. The median level of CMV-CTLs varied considerably for each HLA allele investigated, ranging from 2–30 CMV-CTLs per µl blood (Figure 2A). CMV-CTLs recognizing the HLA-A*2402 tetramer were detected at significantly lower levels in all patients with this HLA molecule compared to CMV-CTLs corresponding to other CMV epitope and tetramer combinations. In addition, 73% of all FACS analyses with the HLA-A*2402 tetramer detected no CMV-CTLs per µl blood, and HLA-A*2402-specific CMV-CTLs did not correlate with the total number of CD3^+^CD8^+^ T cells observed. CMV reactivations did not occur more frequently in patients with HLA-A*2402. In contrast, CMV-CTL levels detected by HLA-A*0101, HLA-A*0201, HLA-B*0702, HLA-B*0801 and HLA-B*3501 tetramers correlated with overall CD3^+^CD8^+^ immune reconstitution (0.5<r<0.8; p<0.0001). The HLA-A*1101-pp65 tetramer was used to monitor only one patient, thus data obtained with this tetramer are not shown in Figure 2.

CMV-reactivation occurred in 42% (n = 117/278) of the patients and had a negative impact on overall survival (p<0.04; Log-rank Test; Figure S1) as expected. After CMV reactivation the median CMV-CTL levels were always higher independent of CMV-epitope tetramer combination used (Figure 2B). The difference in CMV-CTL numbers prior to and after CMV reactivation was statistically significant for HLA-A*0101 (*p*<0.001) and for HLA-A*2402 (*p*<0.01).

**Figure 3:**
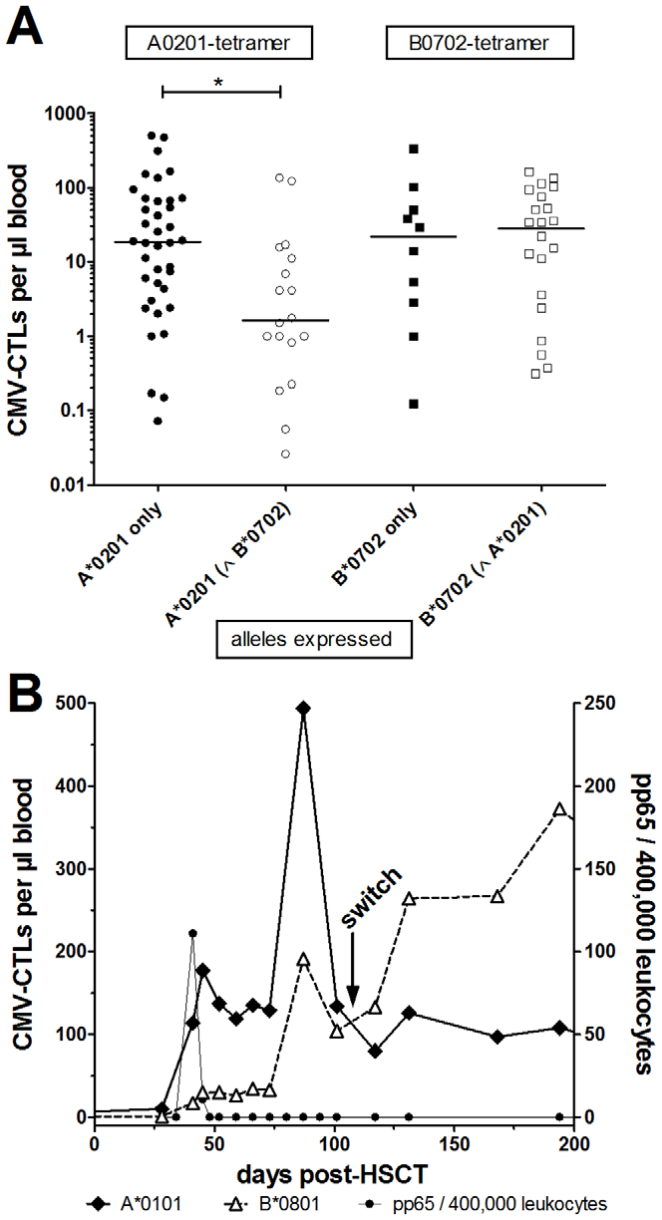
Specific HLA combinations influence the CMV-CTL levels. (**A**) Median CMV-CTL levels in patients expressing only and monitored with HLA-A*0201 (filled circles) or HLA-B*0702 (filled squares) tetramers, respectively, were calculated and compared to the levels in patients expressing both alleles. The level of HLA-A0201 CMV-CTL (white circle) was significantly lower in patients expressing HLA-A0201 and –B0702 compared to those only expressing the A0201 allele (filled circles). No such difference was found for HLA-B0702-binding CTL levels between patients who only express the B0702 allele (filled square) or both A0201 and B0702 alleles (white squares). (**B**) The median numbers of CMV-CTLs detected by each tetramer were compared in all patients monitored with at least 2 different tetramers (n = 176/278). The analysis for one patient is shown as an example. The number of CMV-CTLs detected by each tetramer are plotted over time. CMV reactivation occurred on day +41 (spike in the pp65 line), triggering CMV-CTL expansion. HLA-B*0801 detected the highest numbers of CMV-CTLs until day +100 when a switch occurred. * *p*≤0.05.

Sixty-three percent of the patients (n = 176/278) could be monitored with more than one CMV tetramer (Table 1) and CMV-CTL levels were influenced by the presence of other HLA-tetramer combinations. For example, the level of T cells detected by HLA-A*0201-pp65 was significantly lower, if the HLA molecules expressed by the patients corresponded to both HLA-A*0201 and HLA-B*0702 rather than only the HLA-A*0201 molecule (Figure 3A; median: 1.6 versus 18.5 CMV-CTLs per µl blood; p<0.05; t-Test with Welch's correction). The number of CMV-CTLs detected by HLA-B*0702 did not differ whether the patients also expressed the HLA-A*0201 molecule or not (median: 21.6 and 27.8 CMV-CTLs per µl blood; Figure 3A). A change in the most abundant CMV-CTLs detected with particular tetramers occurred in 11% (n = 19/176) of the patients monitored with at least 2 tetramers. We detected 30 alterations of the most abundant CMV-CTL lines after HSCT. Figure 3B shows a typical example of such a change. This patient experienced CMV reactivation on day +41. A shift from HLA-A*0201 to HLA-B*0801 was detected by day +100, when the level of HLA-B0801 CMV-CTLs rose above the level of HLA-A0201 CMV-CTL. In 14 patients, 17 changes (57%; 17/30) led to higher levels of HLA-B*0801 (IE-1) recognizing CMV-CTLs (Table S2) after day +100 post-HSCT. Interestingly, these increases/decreases of most abundant CMV-CTLs did not correlate with the time of CMV reactivations after HSCT (Table S2).

### Early CMV-CTL reconstitution correlates well with protection against CMV reactivation in the R+/D+ group

**Figure 4:**
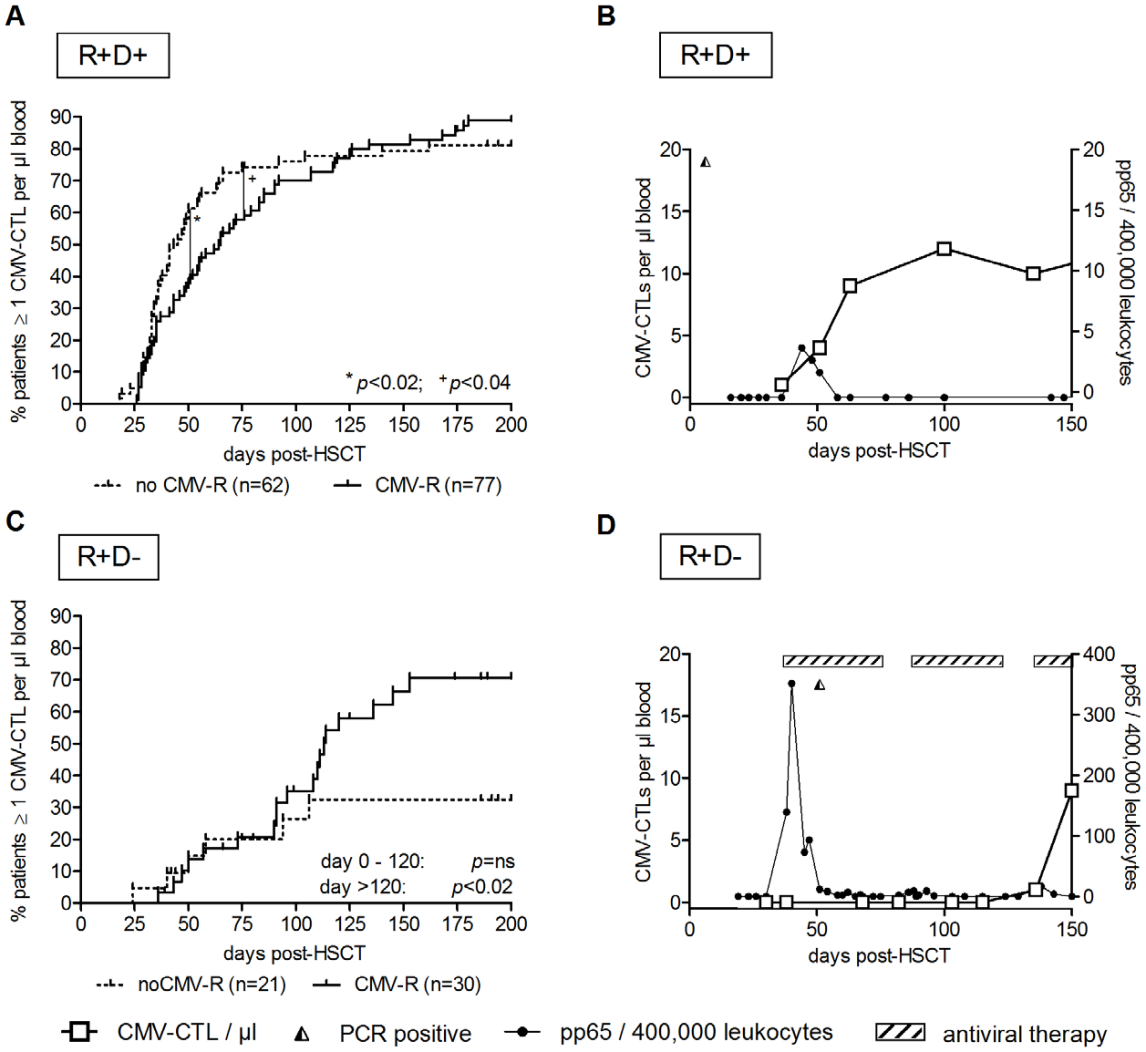
CMV-CTL monitoring enables early discrimination between R+/D+ patients in whom CMV reactivation did or did not occur. (**A**) A threshold of 1 CMV-CTL per µl blood was applied to discriminate between patients with and without CMV reactivation in the R+/D+ group. P-values from log-rank testing are indicated for days +50 (*) and +75 (+). (**B**) CMV-CTL reconstitution after HSCT is shown for one patient in the R+/D+ group. CMV-CTL numbers per µl blood (left y-axis, empty squares) were plotted against the time in days after HSCT. The right y-axis (filled circles) shows the number of pp65-positive cells/400,000 leukocytes as the means of detecting CMV reactivation. Positive PCR results for CMV are indicated at the top of each graph (half filled triangle). CMV reactivation occurred on day +39 (spike in filled circle line) in this patient, after which CMV-CTL expansion occurred (rise in empty square line). (**C**) Reconstitution of CMV-CTLs within the R+D− group using a threshold of 1 CMV-CTL per µl blood. Until day +100 no significant reconstitution occurred. (**D**) Prolonged CMV immune reconstitution (empty squares) and necessary prolonged antiviral therapy (striped bar) in one patient from the R+/D− group.

To identify common features for CMV-CTL reconstitution in our patient cohort, we analyzed the kinetics of CMV-CTL reconstitution. CMV-seropositive recipients (R+, n = 190/278; 68%) were grouped into patients receiving a transplant from a CMV-seropositive donor (R+/D+, n =  139/190) or a seronegative donor (R+/D−, n =  51/190). Monitoring of reconstitution of CMV-CTLs was initiated on day 30 (+/−15 days) post-HSCT in the R+/D+ group. In the R+D+ group sixty two patients had no CMV reactivation after HSCT. By day +50 38 patients (62%, *p*<0.02) had 1 CMV-CTL/µl blood, by day +75 46 patients (74%, *p*<0.04) had achieved 1 CMV-CTL/µl blood. These were significantly more patients than those reactivating CMV at least once (37% by day+50; n = 47/77) (Figure 4A). Interestingly, increasing the threshold level to between 5 and 10 CMV-CTLs per µl blood did not improve discrimination between patients with and without CMV reactivations (Figure S2C–F). A typical example for the R+/D+ patient group illustrates early reconstitution of CMV immunity by day +60 following CMV reactivation (Figure 4B). Patients from the R+/D+ group without detectable CMV-CTLs or with detectable CMV-CTLs that did not expand during or after the first CMV reactivation (12.5% of patients, n = 20). Those were at risk for multiple CMV reactivations (n = 13) similar to patients transplanted from seronegative donors. (R+/D−) showed a delayed reconstitution of CMV immunity, which occurred on or after day +120 (Figure 4C). There is no significant difference in number of patients in the R+/D− group achieving immune reconstitution with 1 CMV-CTL per µl blood with (n = 30) or without (n = 21) CMV reactivation. CMV reactivation was recurrent in all 30 of the 51 patients who did experience reactivation, and required prolonged antiviral therapy. Furthermore, low-level preexisting CMV-CTLs did not proliferate upon CMV reactivation in 27% (n = 14/51) of R+D− patients. As expected, reconstitution of CMV-CTLs was delayed in patients who received TCD grafts, and the course of reconstitution is similar to R+ patients transplanted from CMV-seronegative donors (Figure S3). Patients in the groups R−/D+ or R−/D− were not included in statistical analyses, since patient numbers were small and CMV reactivations (R−/D+ 7/39) or *de novo* infections (R−/D− 3/49) were rare events (Table 1) and did not contribute to the elucidation of CMV reactivation in the context of CMV-CTL reconstitution.

To investigate whether incidence, severity and treatment of aGvHD influenced the numbers of CMV-CTLs detected, we analyzed the associated data for the R+/D+ group. The number of CMV-CTL did not differ significantly in patients without or with aGvHD grade I or II (Figure S4).

### Expansion of CMV-CTLs is associated with single but not multiple CMV reactivations

**Figure 5:**
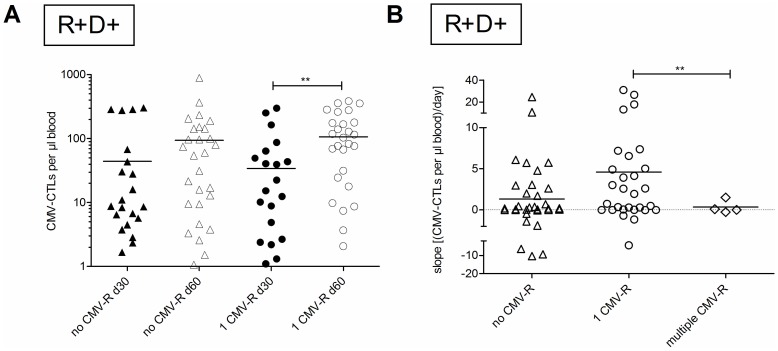
Kinetics of CMV-CTL expansion after CMV reactivation. (**A**) CMV-CTL levels around day +30 (the interval from day +15 to +45 was assessed, filled symbols) and day +60 (the interval from day +46 to +99 was assessed, filled symbols) in patients who experienced (circles) or did not experience (triangles) a CMV reactivation prior to day +100 in the R+/D+ group. Significant differences between groups were assessed by t-test with Welch's correction, and included the 34 data points not depicted because they lie outside axis limits. (**B**) Patients in the R+/D+ group who experienced no (triangles), a single (circles) or multiple (diamonds) CMV reactivations before day +100 were compared (t-test with Welch's correction) using the difference between the slopes of the lines created by the CMV-CTL levels during the interval from day +15 to +45 and the interval from day +46 to +99. The dotted line indicates no change in CMV-CTL level between the measurements in both intervals. ** *p*≤0.01.

CMV-CTL levels detected by the different tetramers (Figure 2) increased after CMV reactivation, thus, defining a time-dependent threshold of CMV-CTL that protects the patient against CMV reactivation is difficult. In our patient cohort, the first CMV reactivation occurred by day +46 (mean; range: +11 to +170) for patients of the R+/D+ group. Thus, we reasoned that protective CMV-CTL levels could probably be predicted by monitoring on days +30 (+/−15) and +60 (+/−15) (Figure 5). Sixty-five of 139 patients of the R+/D+ group were included in these analyses. Figure 5A compares the number of CMV-CTLs on days +30 and +60 in patients who experienced no or only 1 CMV reactivation. CMV-CTLs hardly expanded in patients without CMV reactivation. In contrast, CMV-CTL numbers increased significantly (p<0.004, t-Test with Welch's correction) between days +30 (range: +15 to +45) and +60 (range: +46 to +99) in patients with a single reactivation of CMV. These data illustrate the impact of the first CMV reactivation on CMV-CTL expansion and demonstrate that CMV-CTL levels cannot serve as predictors for the first CMV reactivation. Thus, we assessed whether the proliferation of CMV-CTLs could act as a predictor for a successful restoration of the CMV immune response. We calculated the slope of the CMV-CTL expansion between time-points prior to and after reactivation, and compared CMV-CTL levels in patients without, or with a single or recurrent CMV reactivations (Figure 5B). The expansion slope was significantly higher in R+/D+ patients with only 1 CMV reactivation. In contrast, no significant increase of the slope was seen between days +30 and +60 in patients experiencing recurrent CMV reactivation, indicating that CMV-CTL expansion may be a prerequisite to resolve of CMV reactivation.

## Discussion

CMV reactivation following HSCT is a consequence of the massive immunosuppression and insufficient lymphocyte reconstitution, and occurs more frequently after T cell depletion of the graft or after transplantation of CMV-seropositive patients with grafts from seronegative donors. CMV reactivations still contribute significantly to post-HSCT morbidity, despite advancements made to reduce CMV disease by monitoring for viral reactivation and pre-emptive therapy.

Monitoring CMV-CTL reconstitution can be achieved in about 85% of all transplanted patients [13], [18], using commercially available CMV-tetramers. A large cohort of 278 patients could be monitored with the tetramers, 198 (71%) actually developed detectable CMV-CTLs following HSCT. The CMV-CTL levels detected varied greatly (1–1235 per µl blood), and correlated only weakly with reconstitution of the CD3^+^CD8^+^ T cells, as described previously [23]. Restoration of CMV-specific immunity is frequently analyzed using tetramer staining [14], [15], [24]. However, absolute values for the CMV-CTL levels required to protect the patient from reactivation are still actively debated to date. Our data indicate that CMV-CTL numbers vary considerably for individual combinations of HLA molecules and CMV epitopes. Many authors focus on the HLA allele that is most common in the Caucasian population, HLA-A*0201. Using the HLA-A*0201-NLVP tetramer, 10 to 20 CMV-CTLs per µl blood by day +60 were described as being protective [25], [26]. Our data for CMV-CTL levels detected by HLA-A*0201-NLVP are similar, with a mean level of 10 CMV-CTL per µl blood (Figure 2). However, analyzing additional HLA molecules in our large patient cohort showed that different HLA types yield quite different median values of detectable CMV-CTLs (Figure 2). We and others observed that CMV-CTL levels detected by HLA-A*0101 (pp50_243–255_) or HLA-B*3501 (pp65_123–131_) were considerably higher or much lower than CMV-CTL levels detected by HLA-A*2402 (pp65_341–349_), which yielded a mean of 4 per µl blood (Figure 2) [15], [18], [19], [27]. We detected no CMV-CTLs using the HLA-A*2402 tetramer in 73% of the tests. This is in agreement with data published by others, who detected low levels of CMV-CTLs using the HLA-A*2402 tetramer when monitoring CMV-CTL reconstitution [19], [27], [28], [29], [30], [31], [32]. We also identified no correlation between the immune reconstitution of CD3^+^CD8^+^ T cells and CMV-CTL levels detected using the HLA-A*2402 tetramer. Since CMV reactivation did not occur more frequently in patients expressing the HLA-A*2402 molecule. We speculate that either lower levels of HLA-A*2402-corresponding CMV-CTLs are needed provide protection [19] or the immune-response in HLA-A*2402-positive individuals is dominated by other epitopes [27]. In addition to the different median levels of CMV-CTLs, we found that expression of different HLA alleles may interfere with the expansion of T cells specific for other HLA peptide combinations. For example, in individuals expressing both alleles HLA-A*0201 and HLA-B*0702, the HLA-B*0702-pp65 CMV-CTL response prevailed [33]. Thus, in these patients CMV-CTLs binding to HLA-B*0702-pp65 may be the dominant response to CMV-CTLs recognizing HLA-A*0201-NLVP, since the level of CMV-CTLs binding to HLA-A*0201-NLVP is significantly higher in patients expressing only the HLA-A*0201 allele (Figure 3). Our data imply that there may be more interference between the different HLA molecules and/or CMV epitopes expressed than monitoring techniques currently in use have detected. We documented that changes in the most abundant epitope-specific CMV-CTL population occurred over time. These alterations were solely dependent on particular HLA molecules. Interestingly, in 14 of 19 patients the response directed against the HLA-B*0801 epitope of the immediate early (IE)-1 protein prevailed. We hypothesize that a shift from CMV-CTLs recognizing pp65 epitopes to IE epitopes may delineate the shift from short-term to long-term protection against CMV reactivation [34], [35], [36], [37]. Taken together, the absolute number of CMV-CTLs detected in patient blood samples appears to be depend on the HLA expressed in the patient, the tetramers used for detection of CMV-CTLs and the time-point of the measurement, thus “protective” CMV-CTL levels vary considerably. Taken together, a “protective” CMV-CTL count cannot be defined, since the CMV-CTL level varies a) for the different tetramers used in detection, b) for different HLA combinations expressed in the patient (e.g. A0201 and B0702) and c) for a single tetramer over time. Thus, more information than an absolute number of all CMV-CTLs is necessary to define what is protective against reactivation, and levels defined as protective may vary considerably based on the tetramer or tetramer set used for monitoring.

The most important finding of our study was that significantly more patients who experienced no CMV reactivation had at least 1 CMV-CTL per µl blood before day +75 following HSCT (range: +50 to +75), compared to patients who experienced CMV reactivations (Figure 4). Interestingly, increasing the threshold to 5–10 CMV-CTLs per µl blood did not yield better discrimination between patients experiencing or not experiencing CMV reactivation in our cohort. Lillieri *et al.* and Tormo *et al.* detected similarly low thresholds (1–3 CMV-CTLs per µl blood) using functional assays for IFN-γ and IL-2 secretion or ELISPOT assays [38], [39], [40], implying that, indeed, detection of 1 to 3 CMV-CTLs per µl blood may indicate the hallmark of a functional immunity against CMV.

In addition to the currently controversial CMV-CTL quantity that provides protection against viral reactivation, the influence of CMV reactivations on immune reconstitution is also widely debated. While Chen *et al.* argue that CMV reactivation boosts the reconstitution of CMV-CTLs [25], others find no influence of CMV reactivation on CMV-CTL reconstitution [41]. In our large prospective cohort, patients reactivating CMV (n = 117/278) had higher median CMV-CTL numbers than patients without CMV reactivations (n = 161/278), implying a significant influence of CMV reactivation on levels of CMV-CTLs detected. Since this expansion of CMV-CTLs after CMV reactivation does not allow for the definition of a minimal protective CMV-CTL level, even using only 1 tetramer, we searched for other means to differentiate between patients in whom CMV reactivation occurred only once, was recurrent or did not occur at all. We analyzed CMV-CTLs at two time-points, namely day +30 (+/−15 days) and +60 (+/−15 days), and measured the expansion of CMV-CTLs within that time period. Patients with a protective response after the first CMV reactivation showed a significantly increased expansion of CMV-CTLs within this time period compared to patients with recurrent CMV reactivations. Patients without CMV reactivation also expanded CMV-CTLs during this time interval, but to a lesser extent than those with 1 CMV reactivation. The inability of CMV-CTLs to expand after CMV reactivation may be due to even minor HLA incompatibilities between donor and recipient. Our results indicate that analyses using a single tetramer at only one time point, for instance on day +60, do not allow prediction of pending or, more importantly, recurrent CMV reactivations. Despite the fact that patients without CMV reactivation showed an earlier reconstitution of at least 1 CMV-CTL per µl blood, the wide range of CMV-CTL levels does not allow definition of definitive protective value that is broadly applicable for all HSCT patients. However, monitoring the level of CMV-CTL expansion between days +30 and +60 (+/−15 days) after CMV reactivation can indicate successful restoration of CMV immunity.

In summary, our results show that sequential tetramer monitoring rather single time point cut offs of the post-transplant CMV-CTL immune reconstitution allows a more accurate interpretation of an individual patient's response to CMV. In addition, CMV-CTL expansion after the first CMV reactivation indicates recurrence of CMV reactivation even in R+/D+ patients after allogeneic HSCT. Analysis of the CMV-CTL expansion rate may facilitate implementation of patient-specific antiviral strategies, including adoptive transfer of CMV-CTLs to recipients unable to respond to CMV reactivations.

## Supporting Information

Figure S1 Impact of CMV reactivation on survival. Patients in whom CMV reactivation occurred (n = 117) had a significantly lower probability for survival (*p*<0.04) than patients not experiencing CMV reactivation (n = 161).
(TIF)

Figure S2 CMV-CTL analysis applying different threshold levels. (**A–F**) Reconstitution of CMV-CTLs with respect to reactivation in the R+/D+ patient group using different thresholds of CMV-CTLs per µl blood. Percentage of patients reaching the threshold level is plotted against time after HSCT. (**A–C**) Using thresholds of >0 to ≥3 CMV-CTLs per µl blood showed significant differences between patients with and without CMV reactivation. (**D–F**) Using thresholds of ≥5 or higher (per µl blood) detected no significant differences in the reconstitution of CMV-CTLs between patients with and without CMV reactivation. The asterisk (*) indicates significant differences at day +50; the plus symbol (+) indicates significant differences at day +75.
(TIF)

Figure S3 Impact of T cell depletion on CMV-CTL reconstitution. T cell depletion of the graft results in delayed CMV-CTL reconstitution. CMV-CTL reconstitution in patients receiving T cell depleted (TCD) grafts (continuous line) compared with patients received unmodified grafts (dotted line) after HSCT is plotted against the time in days after HSCT. Significantly fewer patients receiving a TCD-graft reconstituted CMV-CTLs at early time-points following HSCT, however, by day +200 the curves converge. The asterisk (*) indicates significant differences at day +50; the plus symbol (+) indicates significant differences at day +100.
(TIF)

Figure S4 Influence of aGvHD. Median CMV-CTL levels in the R+/D+ group were analyzed with regard to the incidence of aGvHD in the time intervals from day 0 to +49 and days +50 to +99. The mean of the ΣCMV-CTLs is shown for each patient in which CMV-CTL were detected during the interval. (**A**) Patients undergoing CMV-reactivation. (**B**) Patients not experiencing CMV-reactivation.
(TIF)

Table S1 Overview on quality considerations and their applicability to the current cohort. In order to detect tetramer-positive cells at a reliable event rate using the FACS analysis the population requires more than 10 events and the CD8 count must be above 50 per µl blood (using a 200 µl aliquot for staining). Complying with these quality constraints guarantees the theoretical detection of 0.1% tetramer-positive cells (0.05 tetramer-positive cells per µl blood).
(XLS)

Table S2 Switches and clinical characteristics at time of switch. Overview on the 19 patients with switches: patient number, tetramers switching (i.e. which tetramer yielded the higher CMV-CTL per µl blood), time of switch, donor type, CMV-reactivation(s), occurrence of GvHD, application of DLI and relapse are shown. Switches did not correlate with the time clinical events like e.g. CMV-reactivation or DLI.
(XLS)
