# Correction: Sequential Anti-Cytomegalovirus Response Monitoring May Allow Prediction of Cytomegalovirus Reactivation after Allogeneic Stem Cell Transplantation

**DOI:** 10.1371/annotation/43e9b84c-fbe3-4b39-88c8-1cde34b0afea

**Published:** 2013-04-18

**Authors:** Sylvia Borchers, Melanie Bremm, Thomas Lehrnbecher, Elke Dammann, Brigitte Pabst, Benno Wölk, Ruth Esser, Meral Yildiz, Matthias Eder, Michael Stadler, Peter Bader, Hans Martin, Andrea Jarisch, Gisbert Schneider, Thomas Klingebiel, Arnold Ganser, Eva M. Weissinger, Ulrike Koehl

The Acknowledgements section was omitted. It should read:

Acknowledgements

The authors thank W. Kühnau, R. Strasser, S. Luther, U. Lips, O. Grünzel, S. Erben, R. Müller, E. Dirkwinkel, and M. Roth for their excellent technical support, sample collection, sample processing, data collection and data sorting. Further, we thank S. Buchholz and H. Diedrich for their support in clinical issues, M. Morgan and L. Hambach for critical discussion of the manuscript, and B. Held and K. Astrahantseff for manuscript proof-reading. 

