# Supplementary figures and images for: Sequential Anti-Cytomegalovirus Response Monitoring May Allow Prediction of Cytomegalovirus Reactivation after Allogeneic Stem Cell Transplantation
Source: PLoS One. 2012 Dec 13;7(12):e50248. doi: 10.1371/journal.pone.0050248 (PMC3521740; doi:10.1371/journal.pone.0050248)

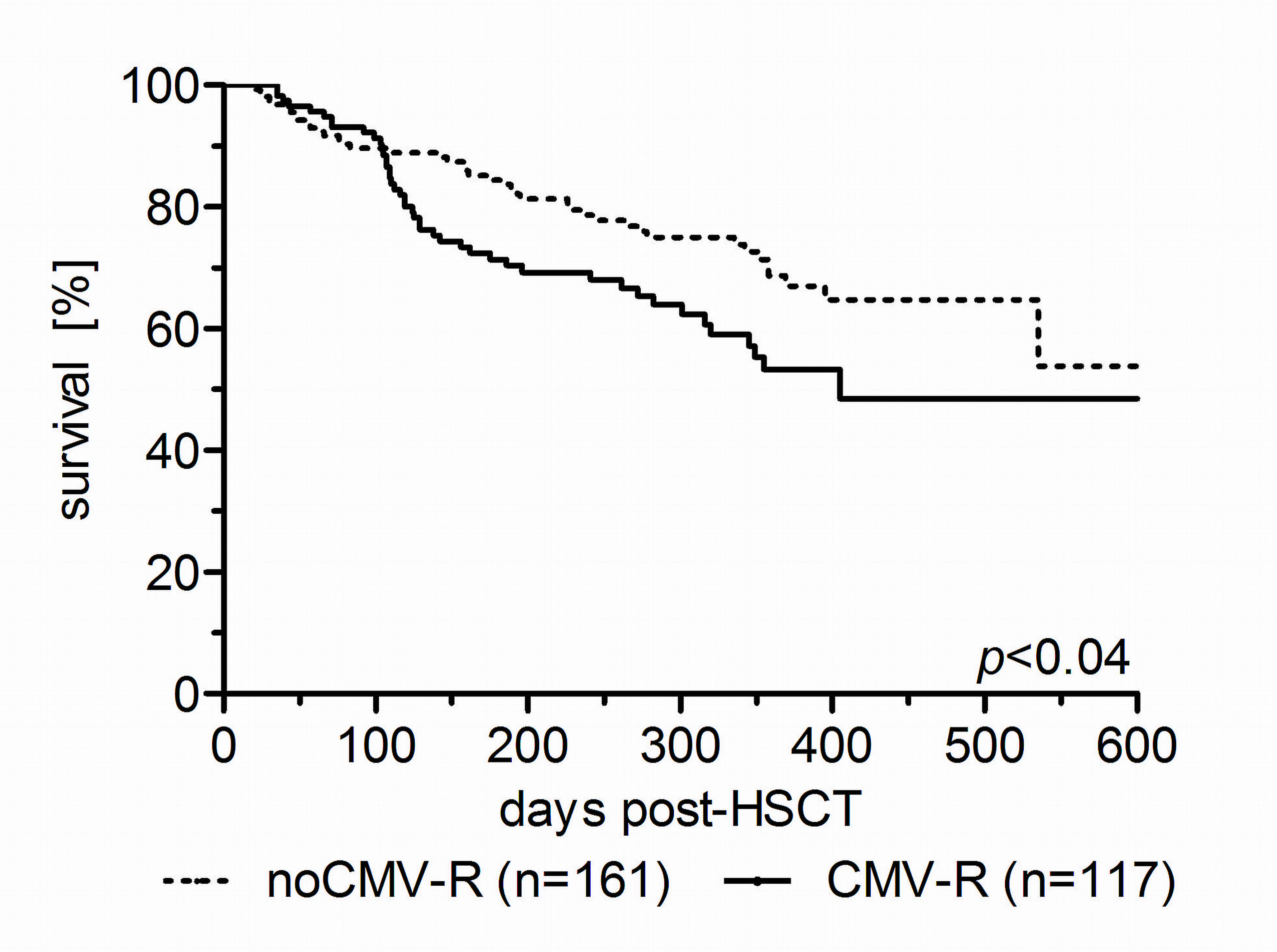

Supplement: Figure S1 — Impact of CMV reactivation on survival. Patients in whom CMV reactivation occurred (n = 117) had a significantly lower probability for survival (p<0.04) than patients not experiencing CMV reactivation (n = 161). (TIF) [file pone.0050248.s001.tif]

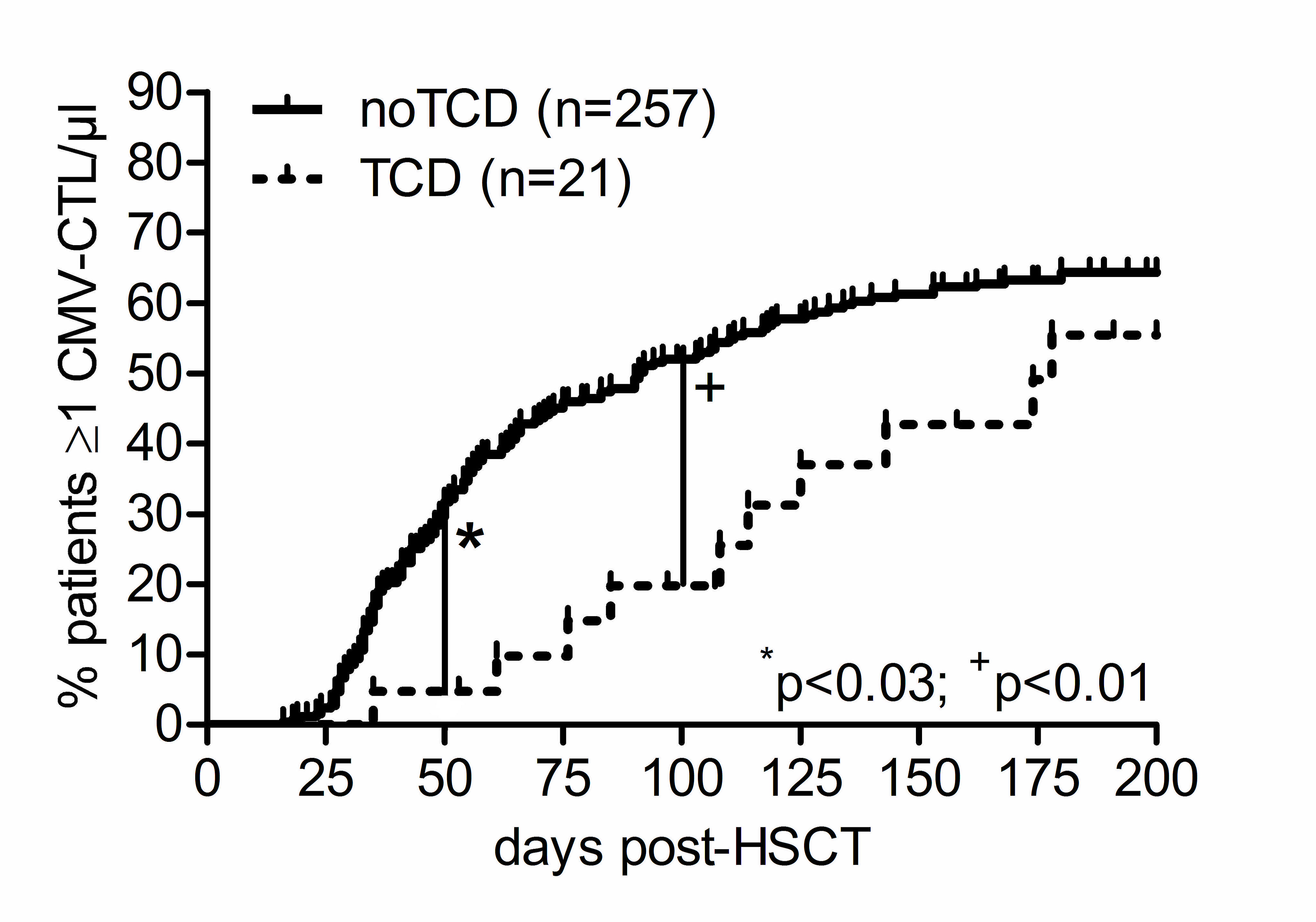

Supplement: Figure S3 — Impact of T cell depletion on CMV-CTL reconstitution. T cell depletion of the graft results in delayed CMV-CTL reconstitution. CMV-CTL reconstitution in patients receiving T cell depleted (TCD) grafts (continuous line) compared with patients received unmodified grafts (dotted line) after HSCT is plotted against the time in days after HSCT. Significantly fewer patients receiving a TCD-graft reconstituted CMV-CTLs at early time-points following HSCT, however, by day +200 the curves converge. The asterisk (*) indicates significant differences at day +50; the plus symbol (+) indicates significant differences at day +100. (TIF) [file pone.0050248.s003.tif]

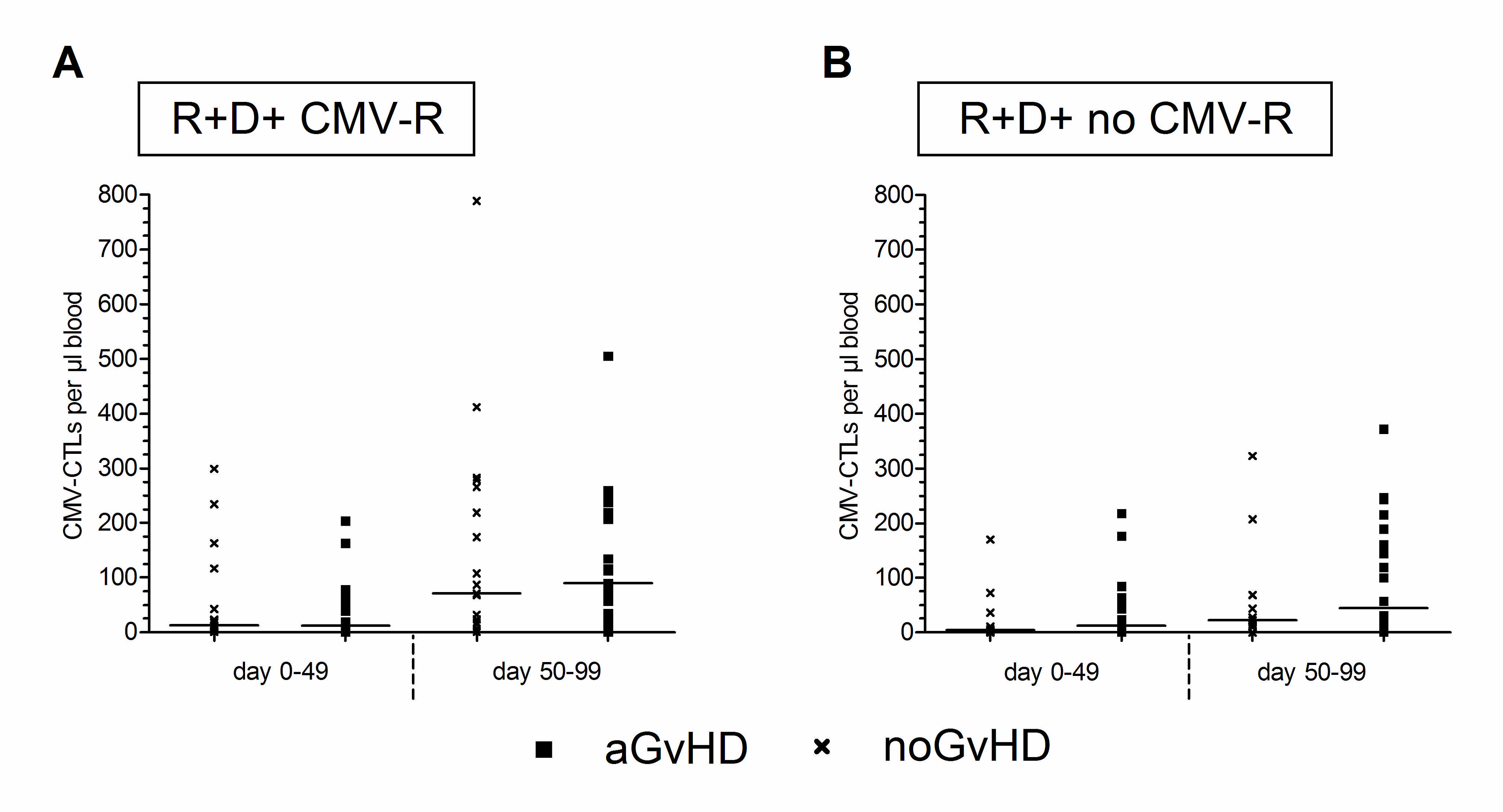

Supplement: Figure S4 — Influence of aGvHD. Median CMV-CTL levels in the R+/D+ group were analyzed with regard to the incidence of aGvHD in the time intervals from day 0 to +49 and days +50 to +99. The mean of the ΣCMV-CTLs is shown for each patient in which CMV-CTL were detected during the interval. (A) Patients undergoing CMV-reactivation. (B) Patients not experiencing CMV-reactivation. (TIF) [file pone.0050248.s004.tif]
